# Electrochemical and theoretical investigations of favipiravir drug performance as ecologically benign corrosion inhibitor for aluminum alloy in acid solution

**DOI:** 10.1038/s41598-023-35226-0

**Published:** 2023-05-29

**Authors:** M. A. Deyab, Omnia A. A. El-Shamy, Hamdy Khamees Thabet, Ashraf M. Ashmawy

**Affiliations:** 1 Egyptian Petroleum Research Institute (EPRI), Nasr City, Cairo, Egypt; 2 Department of Chemistry, College of Sciences and Arts, Northern Border University, PO 840, 91911 Rafha, Saudi Arabia; 3 Chemistry Department, Faculty of Science (Boys), Al-Azhar University, Cairo, 11884 Egypt

**Keywords:** Environmental sciences, Electrochemistry

## Abstract

Aluminum–silicon alloys have become a preferred option in the automotive and aerospace industries thanks to their fault-tolerant process ability and reasonable static characteristics at relatively affordable costs. This study aimed to investigate the use of favipiravir (FAV) drug as a biocompatible and eco-friendly inhibitor to protect aluminum alloy (AlSi) surface in an aggressive acid environment (1.0 M HCl). The electrochemical measurements declare that FAV is categorized as an inhibitor of mixed type with a cathodic effect. At 100 ppm, FAV had the highest inhibitory efficiency (96.45%). FAV is associated with lower double-layer capacitance values and more excellent charge-transfer resistance. These results show that AlSi corrosion in 1.0 M HCl is reduced in the presence of FAV. The Langmuir model is well-suited to the FAV adsorption behavior (R^2^ ≈ 1). Chemisorption is the primary adsorption in this environment. The theoretical calculation studies corrosion inhibitors' molecular structure and behavior. Different quantum chemical properties of the FAV have been calculated, including energy difference (ΔE), softness, global hardness, and energy of back-donation depending on the highest occupied and lowest unoccupied molecular orbitals. In addition, Mulliken and Fukui’s population analysis and the Molecular Electrostatic Potential map represent the electron distribution and the molecule’s active centers. Experimental findings and quantum chemical computations matched, and FAV is recommended as a green corrosion inhibitor.

## Introduction

Aluminum is one of the most flexible and affordable metallic materials for various industries, from soft, highly flexible wrapping foil to the most demanding engineering applications. Due to its excellent casting ability, silicone, an aluminum alloy's primary alloying element, improve aluminum's characteristics. AlSi-based casting alloys comprise around 90% of all aluminum castings^1^. Due to their outstanding wear, low thermal expansion coefficients, high strength/weight ratio, and excellent wear and corrosion resistance, AlSi alloys are widely utilized in different industries, such as the automobile sector, particularly in the production of pistons^2^.

Because most aluminum acid pickling operations in the industry use HCl solution^3,4^, aluminum corrosion is an unavoidable problem affecting practically all chemical businesses and is one of the worst technological disasters of our time. Corrosion is a widespread problem because it unquestionably adds to the deterioration of our natural properties and its direct costs in rupees. Consequently, protecting aluminum and its alloys against hydrochloric acid solutions is critical for expanding industries^5^.

Different organic molecules are used as corrosion inhibitors for metal surfaces^6–9^. Although the higher protection of these organic molecules, most of them cause a harmful environmental effect. The protection of aluminum alloys still needs more concern using characteristic and green effective corrosion resistive materials.

Therefore, corrosion researchers have concentrated on developing of eco-friendly and less toxic corrosion resistance. New corrosion inhibitors with minimal environmental impact, often called green or eco-friendly, have become increasingly desirable and essential^10–12^. The most promising alternative for preventing aluminum from corroding in acidic solutions are drugs since they are typically derived from biological sources, exhibit strong inhibition efficiency at low concentrations, and are naturally biodegradable^13–15^. Hamza et al.^16^ use the weight loss method to examine the phenylephrine drug's adsorption and performance characteristics for corroding Al (2024) alloy in 1.0 M HCl. The authors concluded that increasing the drug concentration improved the efficiency of phenylephrine inhibition. The drug phenylephrine was chemically adsorbed onto the surface, following the adsorption isotherm of Langmuir. At 500 ppm and 303 K, the inhibitor's percentage efficiency was close to 83.92%.

The chemicals in the medications are also biocompatible and created to dissolve effortlessly in the watery environs of the human body because they are made for human ingestion. These characteristics significantly support the medicines' suitability as potential corrosion inhibitors^17^. Pyrazine derivatives are concerned as green and effective corrosion inhibitors^18–20^. FAV is a new antiviral drug that has received approval in Japan to treat pandemic influenza infections that are not yet developed. FAV is a prodrug that is intracellularly ribosylated and phosphorylated to create ibofuranosyl-5-triphos-phate, which is the active metabolite (T-705-RTP). This compound's chemical name is 5-fluoro-2-oxo-1H-pyrazine-3-carboxamide^21,22^. The cost-effectiveness of this kind of inhibitor increases using the expired formula of drugs, as mentioned for other kinds of drugs^17^.

The uses of quantum calculations to study corrosion inhibition have been widely discussed^23–25^. The primary goal of quantum chemistry approaches was to identify and establish links between molecular structure and activity, and a wealth of valuable findings have since been presented^26–28^. Recently, different methods with different basis sets have successfully described the structural significance of corrosion inhibitors and their adsorption performance on the investigated metals^29,30^.

Numerous researchers are interested in the issue of the connection between molecular structure and the effectiveness of the investigated inhibitors^31,32^. The electronic properties of corrosion inhibitors have achieved the appropriate correlation, such as their highest occupied and lowest unoccupied molecular orbitals, energy difference, electronegativity, atomic charge, and dipole moments. Recently, theoretical calculations^33,34^ are successfully applied to relate the chemical structure of the inhibitors and their adsorption efficiency on the surface of the metal.

The goal of this paper is electrochemical evaluation for the inhibition efficiency of favipiravir (5-fluoro-2-oxo-1H-pyrazine-3-carboxamide) as a new corrosion inhibitor for aluminum alloys in the most suitable aggressive medium (HCl). The adsorption behavior of the applied drug was investigated and discussed. In addition, Quantum chemical descriptors are calculated, and correlate the electronic characters with the experimental data.

## Experimental

### Material

**Figure 1:**
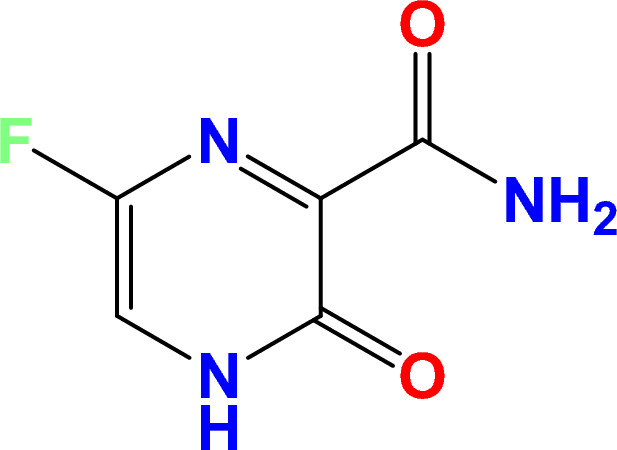
Structure for favipiravir (5-fluoro-2-oxo-1H- pyrazine-3-carboxamide).

BDR Pharmaceuticals International Pvt. Ltd. provided favipiravir (5-fluoro-2-oxo-1H-pyrazine-3-carboxamide) (FAV) (99.98%) (Mumbai, India). Aluminum silicon alloys (AlSi) obtained from GEST for the metal company possess the following weight composition (in percentages): Cu-1.99, Si-9.89, Mn-0.22, Ni-0.269, Zn-2.44, Cr-0.037, Fe-1.10, Ca-0.004, and Al-balance used in the experiment. The inhibitor used can be drawn in the following (see Fig. 1).

### Corrosion medium

With distilled water and a 37% HCl(AR grade) solution, the aggressive aqueous medium (1.0 M HCl) was prepared. FAV concentrations range from 20 to 100 ppm. FAV is utterly soluble in1.0 M HCl solution without solvent.

### The electrochemical studies

A three-electrode electrochemical unit with AlSi as the anodes and the reference and counter electrodes, a saturated calomel electrode (SCE), and a platinum coil, respectively, was utilized in the experiments. At 1.0 mVs^−1^ (scan rate), the potentiodynamic curves were moved from −0.30 to +0.30 V (SCE) concerning OCP. After dipping the anode in the test for 600 s, in the frequency range of 100 kHz–10 mHz, electrochemical impedance spectroscopy (EIS) was achieved at OCP with a voltage perturbation (10 mV). These methods were tested using the Gamry3000 “potentiostat/galvanostat/ZRA”, and the data was analyzed using Echem Analyst 7.

For each concentration, three replicate experiments were performed. The standard deviation and mean values for the inhibition performance measurements of the FAV were shown statistically.

### Quantum chemical calculation

Using the Materials Studio v7.0 DMol3 calculation model, the investigated molecules were constructed and geometrically optimized using the DNP basis set. Quantum chemical parameters such as Frontier molecular orbital, “the highest occupied (E_HOMO_) and lowest unoccupied (E_LUMO_) molecular orbitals, energy difference “ΔE”, ionization potential "I”, electron affinity “A”, electronegativity “χ”, softness “σ”, and global hardness “η” were obtained using the next relations^28,35^.

1 $$\mathrm{\Delta E}={\mathrm{E}}_{\mathrm{LUMO}}-{\mathrm{E}}_{\mathrm{HOMO}}$$

2 $$\mathrm{I}=-{\mathrm{E}}_{\mathrm{HOMO}}$$

3 $$\mathrm{A}=-{\mathrm{E}}_{\mathrm{LUMO}}$$

4 $$\upchi =\frac{\mathrm{I}+\mathrm{A}}{2}$$

5 $$\upeta = \frac{\mathrm{I }-\mathrm{ A}}{2}$$

6 $$\upsigma = \frac{1}{\upeta }$$

### Adsorption annealing simulations

The inhibitor's molecule orientation on the metallic surface was determined using adsorption annealing simulations. Using a fractional thickness of 7.014 and a cleave plane of 111, one can split a bulk cubic Al unit cell to produce the adsorbate surface. For the purpose of removing any potential interactions between the periodic images of the system, a 6 × 6 × 1 supercell was constructed and repeatedly copied in three dimensions with at least 20 mm of vacuum between each copy. Condensed-phase optimized molecular potentials for atomistic simulation studies (COMPASS), a second-generation force field that makes precise thermo-physical property predictions for a range of materials possible, were used depending in Gaussian 09 to add a geometry-optimized FAV onto the metal alloy surface using the adsorption locator included in the Biovia Materials Studio package^36^.

## Result and discussion

### An investigation into the electrochemistry

#### Potentiodynamic polarization measurements

**Figure 2:**
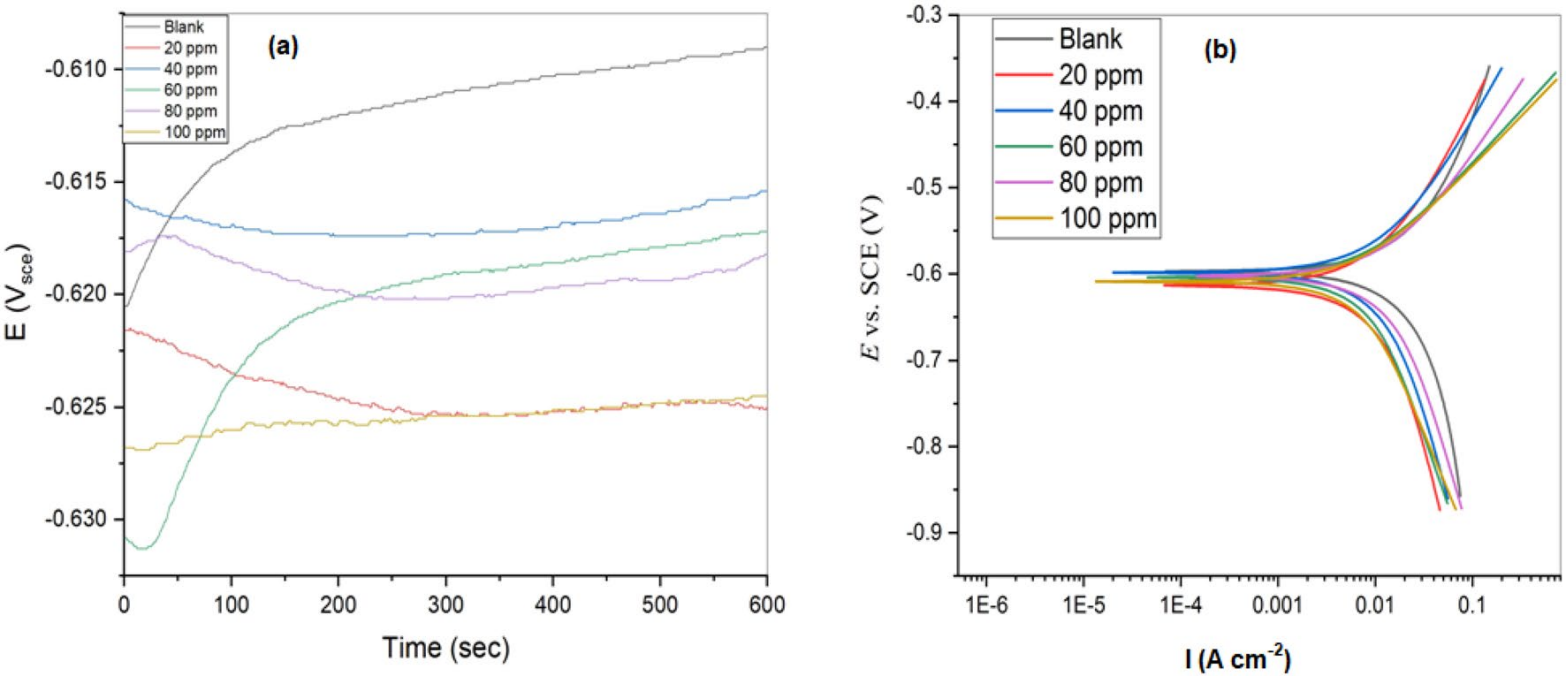
(**a**) Plot of OCP against time and (**b**) Polarization profiles for AlSi in the absence and presence of different doses of FAV in 1.0 M HCl at 298 K.

The plot of OCP against time for AlSi in 1.0 M HCl solution in the absence and presence of different doses of FAV at 298 K is shown in Fig. 2a. After 600 s, the OCP became stable and steady values.

Figure 2b declares the pattern of both cathodic and anodic polarization in 1.0 M HCl for AlSi at 298 K in the absence/presence of various dosages of FAV. The utilization of Tafel lines to quantify electrochemical kinetic descriptors involving corrosion potential (E_corr_), Tafel slopes (B_a_ & B_c_), and the current density of corrosion (I_corr_) (see Table 1). The I_corr_ represents the corrosion rate for electrochemical corrosion^37^. FAV's inhibitory efficiency (*E*_P_%) is computed using^38^:

7 $${\text{E}}_{{\text{P}}} \% = \frac{{{\text{I}}_{{{\text{corr}}\left( 0 \right)}} - {\text{I}}_{{{\text{corr}}}} }}{{{\text{I}}_{{{\text{corr}}\left( 0 \right)}} }} \times 100$$

where I_corr(0)_: the current density of corrosion for blank.

**Table 1:** Corrosion parameters obtained from potentiodynamic polarization measurements of AlSi in the absence and presence of different doses of FAV in 1.0 M HCl at 298 K.

Solution	Conc. ppm	− E_corr_ mV vs SCE	I_corr_ (μA cm^−2^)	Β_a_ (mV dec^−1^)	β_c_ (mV dec^−1^)	θ	*E*_P_%
Blank	0	597 ± 4	96.4 ± 3.6	655	3061		
FAV	20	599 ± 3	17.2 ± 1.6	247	508	0.8251	82.15
	40	614 ± 4	15.6 ± 1.2	261	540	0.8381	83.81
	60	604 ± 6	12.0 ± 1.3	175	423	0.8755	87.55
	80	603 ± 4	8.55 ± 0.89	80	229	0.9113	91.13
	100	609 ± 5	3.42 ± 0.34	47	151	0.9645	96.45

Concerning Table 1, the results indicate that adding FAV causes a significant reduction in the current density of I_corr_. When FAV is introduced to 1.0 M HCl, the anodic metal dissolving and cathodic reduction processes are slowed. Additional examination reveals that the impact of suppressing anodic AlSi dissolving reaction is less pronounced than the effect of hindering the cathodic reduction process.

Table 1 demonstrates that adding FAV only slightly changes the cathodic direction of E_corr_ values. When E_corr_ varies by more than 85 mV, the inhibitor is classified as cathodic or anodic^39^. E_corr_ is shifting by about 17 mV compared to a blank solution (Table 1), indicating that FAV is an inhibitor of mixed types.

The addition of FAV resulted in considerable changes in the values B_a_ and B_c_, as shown in Table 1. The change in B_a_ readings was connected to the potential of a redox complexation process involving AlSi–FAV complexes, and it was also influenced by the amount of the FAV^40^. The inhibitory effectiveness improves as the FAV concentration rises from 20 to 100 ppm. The maximal inhibition efficiency (*E*_P_% = 96.45) is attained at 100 ppm, indicating that FAV is an effective AlSi inhibitor (1.0 M HCl). FAV’s corrosion efficiency does not change significantly above 100 ppm. This is most important due to the forming of an adsorptive and resistive coating of FAV on the surface of AlSi^41^. The layer is a physical barrier, preventing corrosive species from diffusing to the AlSi surface. The FAV adsorbed on the surface of AlSi due to the existence of oxygen and nitrogen atoms. The oxygen and nitrogen atoms are thought to be the adsorption process's reaction center point. FAV adsorption on an AlSi surface typically entails one or more H_2_O molecules that have been adsorbed on the AlSi surface being swapped out.

#### Electrochemical impedance measurements (EIS)

**Figure 3:**
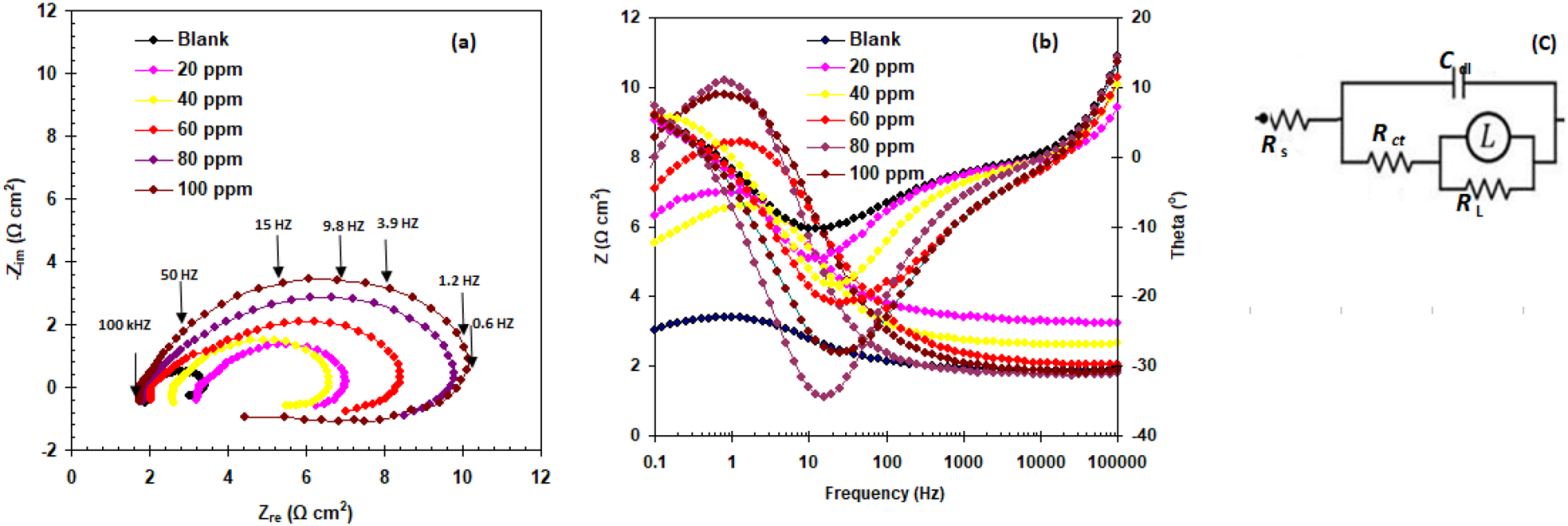
Impedance spectra (**a**) Nyquist, (**b**) Bode-module and phase angle plots, (**c**) equivalent circuit for AlSi in the absence and presence of different doses of FAV in 1.0 M HCl at 298 K.

To gather information regarding the surface active layers on the AlSi, an EIS study in 1 M HCl was performed without and with various FAV dosages. Figure 3a depicts the appropriate Nyquist graph, and Fig. 3b depict the Bode graphs of the AlSi that studied at 298 K. The Nyquist graphs depict a capacitive loop operating at a very high frequency (HF) and an inductive circuit operating at a low frequency (LF). The resistance of charge transfer of the oxide layer on Al might be attributed to the HF capacitive loop^42,43^. An inductive circuit was also responsible for the dissolution of Al at low frequencies and the re-dissolution of the surface oxide film. Surface area variation or salt film property modification, such as density, ionic conductivity, or thickness, can explain inductive behavior. The diameter of HF and LF loops rose noticeably as the FAV amount increased. This might be due to the formation of a film on the face of the AlSi alloy^44,45^.

The plot of Z versus Frequency in Fig. 3b demonstrates that the impedance, which is connected to the charge transfer resistance, *R*_ct_, increases as the FAV concentration rises. Due to their polar nature, the high concentration of FAV molecules in the solution tends to adhere to the AlSi alloy surface and interact with one another to form a layered assembly capable of preventing electron charge transfer, which is what causes the |Z| increment.

Additionally, it is noted that the presence of the FAV causes the medium frequency phase maximum (ɵ_max_) to increase (see Fig. 3b). A more effective barrier impact by the FAV layers towards corrosive ions is inferred by the increase in ɵ_max_ because the medium frequency behavior is caused by diffusion through the surface films.

Fitting EIS data to an equivalent electrical circuit model is shown in Fig. 3c.

The EIS impedance data were recorded in Table 2. FAV increases charge-transfer resistance (*R*_ct_) while decreasing double-layer capacitance (*C*_dl_). These results demonstrate that the including of the FAV compound minimizes the corrosion of AlSi in 1.0 M HCl. The inhibitory power of FAV is calculated from EIS data (*E*_R_%) employing the formulas below^46^:

8 $$E_{{\text{R}}} \% = \frac{{R_{{{\text{ct}}}} - R_{{{\text{cto}}}} }}{{R_{{{\text{ct}}}} }} \times 100$$

where *R*_cto :_ the charge-transfer resistance for blank.

**Table 2:** EIS parameters of of AlSi in the absence and presence of different doses of FAV in 1.0 M HCl at 298 K.

Solution	Conc. ppm	*R*_s_ (Ω cm^2^)	*R*_ct_ (Ω cm^2^)	*C*_dl_ × 10^−3^ (F cm^−2^)	Y_o_ (μ Ω ^−1^ s^n^ cm^−2^) × 10^−3^	θ	*E*_R_%
Blank	0	1.95 ± 0.23	1.28 ± 0.15	7.55 ± 0.35	5.89	–	–
FAV	20	3.42 ± 0.19	3.26 ± 0.21	3.72 ± 0.26	4.09	0.6065	60.65
	40	2.73 ± 0.24	3.40 ± 0.24	2.38 ± 0.15	6.41	0.6229	62.29
	60	2.25 ± 0.16	5.28 ± 0.28	1.13 ± 0.10	1.77	0.7571	75.71
	80	1.85 ± 0.14	5.84 ± 0.32	2.36 ± 0.17	3.36	0.7805	78.05
	100	2.0 ± 0.11	6.85 ± 0.39	1.43 ± 0.14	1.85	0.8129	81.29

The readings of *E*_R_% rise with FAV dosage (Table 2). At high doses 100 ppm), the *E*_R_% of FAV became 81.29%. The efficiency pattern in impedance analysis is analogous to that in polarization analysis.

### Adsorption isotherm

An adsorption isotherm is helpful for figuring out the forms, locations, and interactions between a metal surface and an inhibitor. The primary parameters that explain the behavior of an inhibitor in a corrosive medium on the surface of metal are θ surface coverage and (C) the inhibitor concentration. The Langmuir models were applied to characterize the adsorption process and interactions between metal surfaces and PAV molecules. The slope, intercept, and regression coefficient are determined by applying the abovementioned models and plotting the values using a linear equation. The Langmuir model has the best R^2^ values, approximately equal to 1. Therefore, we expected the AlSi surface to have several active corrosion sites, each covered by one adsorbed molecule^47^.

**Figure 4:**
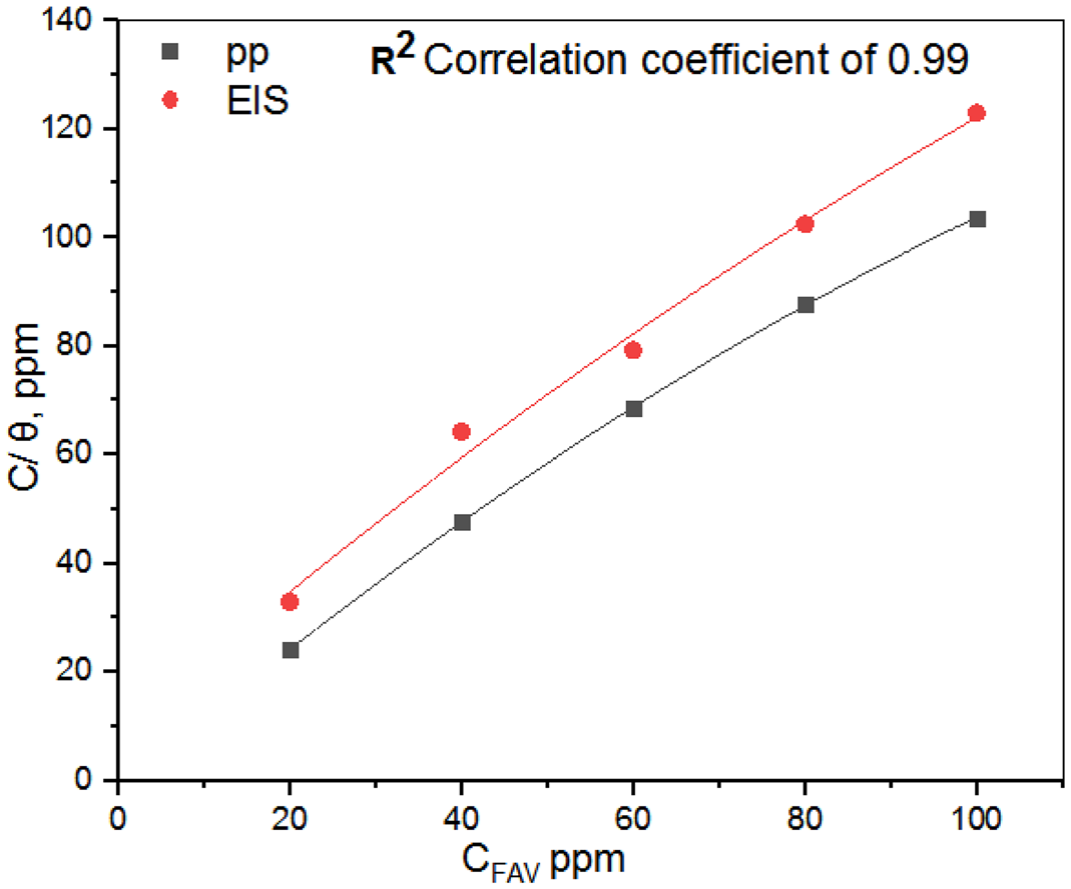
Langmuir adsorption isotherms for FAV at 298 K, obtained from PP, EIS data.

The relationship between the concentration (C_FAV_) and surface coverage (*θ)* of inhibitors in the Langmuir model expressed as following:

9 $$C_{FAV} /\theta = 1/K_{ads} . + C_{{{\text{FAV}}}}$$

where K_ads_: the equilibrium constant and *C*_FAV_ : the concentration of the inhibitor by substitution in the above equation with the values of surface coverage (*θ*) obtained from PP and EIS measurement and then plotting a relation between *C*_FAV_/$\theta$ and *C*_FAV_ we can calculate the values of K_ads_, Fig. 4. The calculated values of *K*_ads_ of the inhibitor are 0.014592, and 0.012618 M^−1^ for PP and EIS respectively. The higher values of *K*_ads_ indicate strong adsorption of the inhibitor on the surface of AlSi alloy^48,49^. The standard free energy of adsorption ΔG^0^_ads_ were obtained using the next relation^9,50^:

10 $$\Delta {\text{G}}_{{{\text{ads}}}}^{0} = - {\text{ RT ln}}\left( {{55}.{\text{5 K}}_{{{\text{ads}}}} } \right)$$

The concentration of water molecules in the solution (in mole/l) is represented by the factor 55.5, and R: the universal gas constant (8.314 J/Kmol). Negative ΔG^0^_ads_ indicates that FAV adsorption is a thermodynamically spontaneous process that can form a stable, protective layer on the Al Si alloy surface. In general, if ΔG^0^_ads_ has an absolute value of less than 20.0 kJ/ mol. The adsorption process is physisorption; however, if the adsorption energy is < 40.0 kJ/mol, the adsorption is chemisorption. pp has a value of − 58.5653 kJ/mol, while EIS has a value of − 54.8825 kJ/mol. So, the main adsorption, in this case, is chemisorption^51,52^.

### Quantum chemical calculation

**Figure 5:**
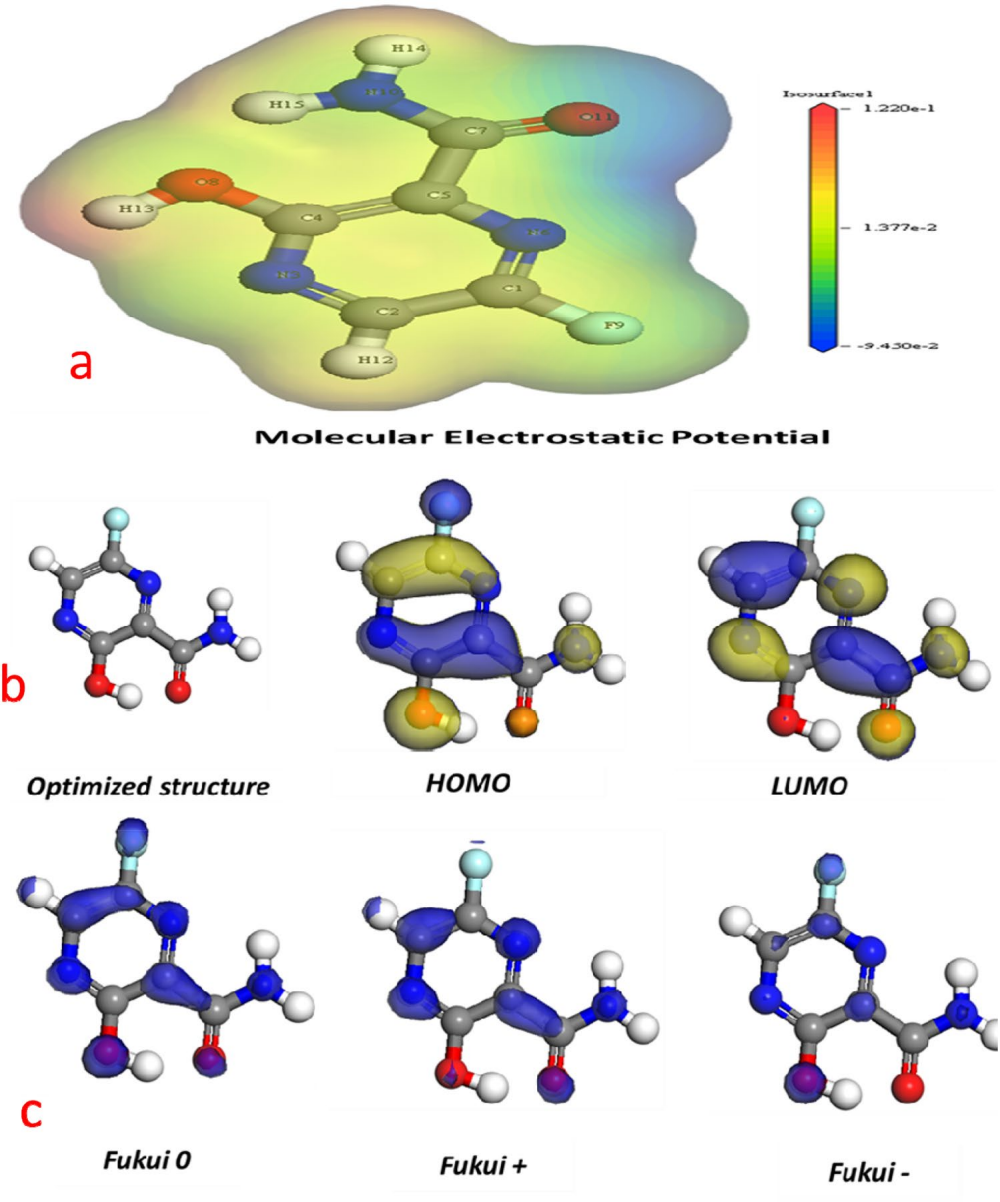
(**a**) Molecular electrostatic potential (**b**)optimized, HOMO, and LUMO for FAV. (**c**) Fukui functions for FAV.

computational calculations are usually study corrosion inhibition mechanism without using lab instruments^53^. Using these results, it is possible to suggest a method of correlation between FAV and Aluminum alloys. The reactivity of inhibitors has been visualized using Molecular Electrostatic Potential (MEP) based on the colors of the area for nucleophilic and electrophilic attacks. The MEP maps of both the protonated and neutral inhibitors were show in Fig. 5a. The colors red and green on the MEP map are vulnerable to nucleophilic attack, while green and blue are vulnerable to electrophilic attack^54^.

After Geometry optimization (total energy =  − 640 eV), HOMO (the electron donation ability) and LUMO (back-donation ability) distributions of the inhibitor are obtained and represented in Fig. 5b. The HOMO localized over the rich electron density part of FAV (benzene ring and oxygen). In addition, LUMO located on the entire molecule. Concerning Table 3, FAV possesses lower energy difference and EHOMO and a small value of ELUMO, indicating its absorption onto AlSi surface via donation and back-donation^28,55^. In addition, a lower value of energy gap (ΔE), electronegativity, and ionization potential (see Table 3) facilitate the tendency of the FVA to donate an electron to the vacant orbital of the investigated alloy. Dipole moment (μ) measure of how hydrophobic a molecule is, and the results indicate that lower dipole moment molecules are more conducive to the adsorption efficiency of the inhibitor molecule on the metal surface^32^. The energy shift related to the back-donate interaction is calculated by Ebenso et al.^56^ depending on the global hardness (ΔE_back-donation_ =  − η/4). For FAV, the negative energy values indicate that the donation and back-donation process is energetically or spontaneously favored. FAV’s lower value of Dipole moment (μ = 1.65 Debye) confirmed its adsorption efficiency. Furthermore, other important quantum descriptors, such as global hardness (η) and higher softness (σ) are calculated and prove the significant reactivity of FAV^57^.

**Table 3:** Quantum-chemical parameters for FAV compound.

Parameters	E_Homo_ (ev)	E_Lumo_ (ev)	ΔE (ev)	I (ev)	A (ev)	Χ (ev)	η (ev)	σ (ev)	(ΔE_back-donation_) (ev)
FAV	− 0.24	− 0.13	0.11	0.24	0.13	0.19	0.06	16.66	− 0.015

Concerning the MEP map and the previously calculated quantum descriptors, the investigated drug possesses nucleophilic character with less electrophilic sites, indicating the great tendency of adsorption to the metal surface with supported back donation. The previous electronic properties are matched with the experimental data, and both confirmed that ability of the FAV for donation and back donation to form a resistive layer on the metal surface.

Furthermore, Mulliken and Fukui population analysis may be used to calculate atomic charges, and these calculations may be useful in understanding the molecular characteristics of FAV molecules. These directories are generated using the Dmol_3_ module in the Materials Studio 7 software. Fukui indices are a critical indicator that shows the local reactivity of a chemical. As a result, it's critical to conduct a detailed examination of atomic sites to understand better how local reactive sites and inhibitory effects are related. Also, defining these atomic sites from a molecule's reactivity perspective allows these hypotheses to be linked^58^.

The condensed Fukui functions presented Fig.5c for both the nucleophilic(f+) and electrophilic (f−) attacks, in addition to the electrophilic attack on the N atom. Calculated Mulliken atomic charges for each compound's atoms show that nitrogen and oxygen atoms are often the more electronegative. Figure 5c shows the condensed Fukui functions for both (f^+^) and (f^−^) attacks, as well as the electrophilic attack on the N atom. Calculating Mulliken atomic charges for each compound's atoms demonstrates that the most electronegative atoms are N and O.

### Adsorption annealing simulations

**Figure 6:**
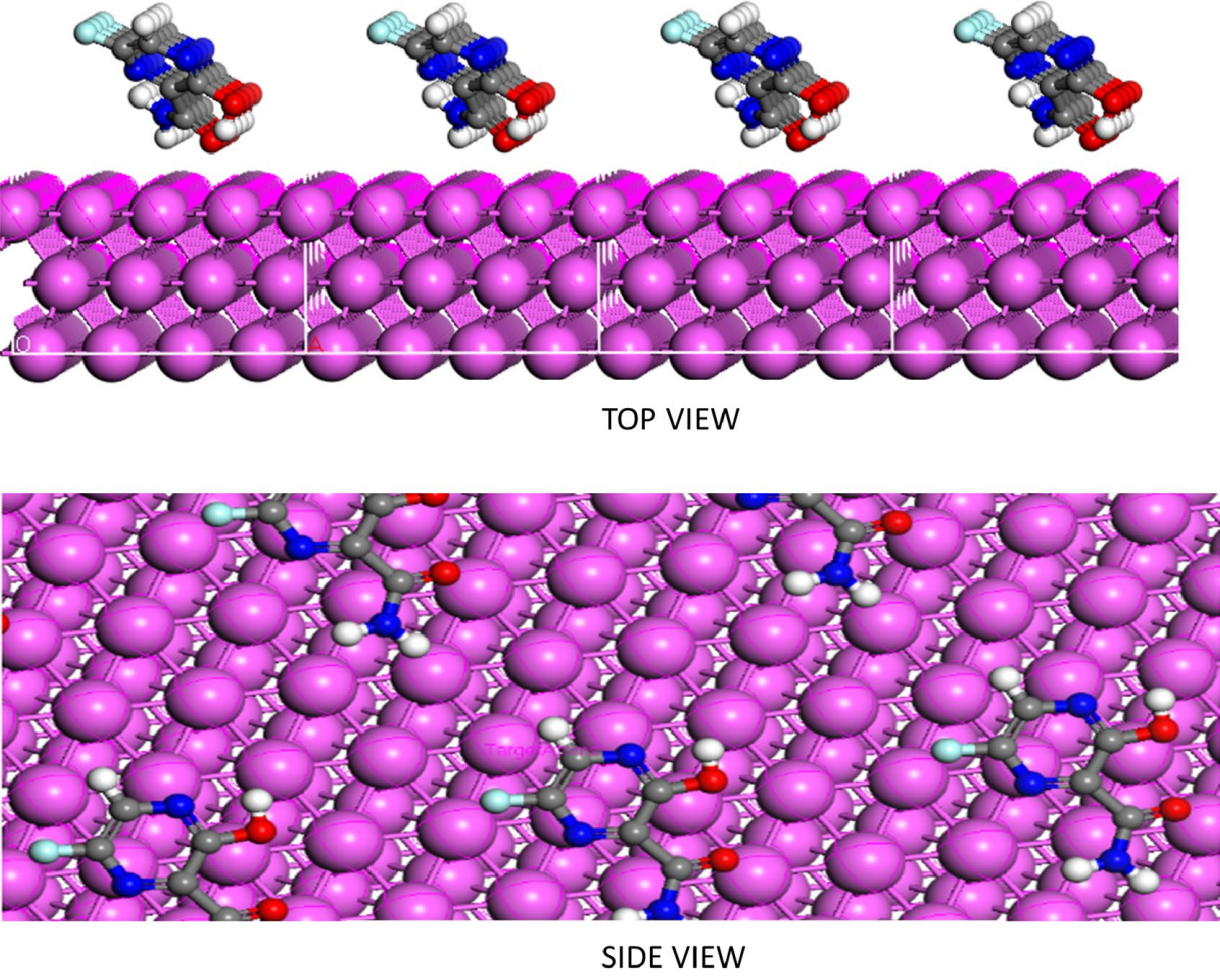
Side and top views of the most appropriate configuration for adsorption of FAV on Al (110) surface obtained by MD simulations in the aqueous solution.

Figure 6 shows the FAV’s adsorption anneals onto the surface of AlSi. FAV has the adsorption energy during the simulation process, confirming the inhibition efficiency of FAV [111] (see Table 4). As a result, the FAV molecules are adsorbed on the AlSi surface, generating stable adsorbed layers that provide corrosion protection for the AlSi surface from 1.0 M HCl, as both practical and theoretical studies have shown. In this scenario, the presence of an amide group provides a more remarkable ability to interact with the AlSi surface. As a result, it is undeniably true that the existence of heteroatoms and electron-donating groups, as well as the delocalization of the π-electron, facilitate the interaction and degree of adsorption of the examined FAV. Table 4 summarizes the interaction and binding energy of the investigated inhibitor. The FAV has a higher binding energy (212.28 kcal/mol) compared to by other drugs, antipyrine derivatives^58^ and antifungal substances that was applied as green inhibitors for aluminum alloy (Bifonazole (B.E. =  − 76.31), Econazole (B.E. =  − 76.31), and Butoconazole (B.E. =  − 76.31))^59^. This confirmed that FAV has a greater chance of adhering to metal surfaces and is more efficacious^60^.

**Table 4:** Calculated parameters from adsorption annealing simulation of the adsorbate onto the adsorbent Al(111) surface.

Parameters	I
Adsorption energy kcal/mol	212.28
Rigid adsorption energy kcal/mol	− 56.57
Deformation energy kcal/mol	− 155.72
Adsorbate: (dE_ads_/dNi) kcal/mol	− 212.28
Binding energy kcal/mol	212.28

## Conclusions

To assess the observed inhibitory effects and throw additional light on how the favipiravir drug inhibits corrosion and interacts with metal surfaces, quantum chemical calculations and electrochemical measurements were investigated. From the research, the following findings can be drawn:

1. FAV drug show excellent corrosion inhibition for aluminum alloys (AlSi) in an acid environment.
2. The inhibition efficiency of the investigated drug increase with concentration, reaching a maximum performance of 96.45% at 100 ppm.
3. Polarization and EIS experiments demonstrated that FAV considerably influences the rate of AlSi corrosion and acts as a mixed-type inhibitor.
4. The Langmuir isotherm governs FAV adsorption on AlSi surfaces.
5. The adsorption of FAV on the AlSi surface is spontaneous with chemicals in nature.
6. FAV possesses a higher binding energy = 212 kcal/mol, indicating that it is more likely to be adsorbed on metal surfaces and is more potent than the previously listed drugs.
7. The corrosion parameters determined from the experimental and the quantum computations correspond well.

## Data Availability

The datasets used and/or analyzed during the current study available from the corresponding author on reasonable request.
